# *GoniOwl*: convolutional neural network-based sample state detection for collision prevention on synchrotron beamlines

**DOI:** 10.1107/S1600577526007277

**Published:** 2026-08-07

**Authors:** Christian M. Orr, James O’Hea, Armin Wagner

**Affiliations:** a Diamond Light Source Ltd Harwell Science and Innovation Campus Didcot United Kingdom; RIKEN SPring-8 Center, Japan

**Keywords:** collision prevention, sample changers, image classification, convolutional neural networks, beamline automation, macromolecular crystallography, *EPICS*, *TANGO*, machine-protection systems

## Abstract

Preventing collisions during automated sample exchange is critical for synchrotron beamlines, particularly for complex cryogenic in-vacuum end­stations where recovery from hardware damage may take days. *GoniOwl*, a compact convolutional neural network (CNN) model, classifies sample-pin presence on the goniometer from a live camera feed on the long-wavelength macromolecular crystallography beamline I23 at Diamond Light Source. Trained on over 8700 manually verified images spanning two years of routine operation and augmented for robustness to illumination changes, camera shifts and occlusions, the model achieves >99% accuracy with millisecond-level inference. A confidence-gating mechanism routes uncertain predictions to a fail-safe path requiring operator confirmation, ensuring suitability for machine-protection control. Integrated via *Experimental Physics and Industrial Control System* (*EPICS*) process variables, *GoniOwl* runs in real time within the automated sample-change sequence. In shadow-mode deployment, the CNN matched or exceeded both the legacy histogram method and operator confirmations, which each achieved 96% accuracy. A closed-loop disagreement-audit workflow automatically collects divergent cases for targeted retraining and verification. The approach is readily transferable to other beamline environments where camera-based vision systems can provide an additional software machine protection layer.

## Abstract 2

*GoniOwl*, a lightweight convolutional neural network integrated into the *EPICS* control system, detects sample-pin presence from beamline camera images with >99% accuracy and fail-safe uncertainty handling, offering a transferable approach to vision-based collision prevention on synchrotron beamlines.

## 1. Introduction

Synchrotron beamlines are complex experimental environments where precise mechanical coordination is essential (Rees *et al.*, 2007 ▸). Collisions between components such as goniometers, sample changers and detectors can cause costly damage, disrupt user schedules and compromise experimental outcomes. As facilities move towards higher automation and throughput, the need for reliable autonomous collision-prevention systems has become critical (Nurizzo *et al.*, 2016 ▸; O’Hea *et al.*, 2017 ▸). Large cryogenic in-vacuum sample environments, like the endstation of the long-wavelength macromolecular crystallography beamline I23 at Diamond Light Source (Wagner *et al.*, 2016 ▸; Duman *et al.*, 2021 ▸), require robust systems, as interventions to recover from collisions can take days to weeks.

Current strategies include mathematical models (Grama & Wagner, 2016 ▸), hardware interlocks and vision-based methods such as histogram analysis. While these approaches provide partial safeguards, they suffer from limitations: mathematical models require perfect calibration, hardware interlocks lack flexibility, and histogram-based techniques are sensitive to lighting changes, camera shifts, reflections and occlusions. Logical tracking systems and digital twins offer additional layers of protection but remain vulnerable to unexpected user actions and real-world variability (Sulaiman Khail *et al.*, 2023 ▸). These constraints highlight the need for a more adaptive and robust solution.

In this work, we introduce a convolutional neural network (CNN)-based image classification model, *GoniOwl*, that determines whether a sample pin is present on the goniometer using a live camera feed. Unlike traditional histogram methods, our approach is resilient to operational variability and supports autonomous operation, with fail-safe operator intervention only for uncertain cases. Trained on a large dataset of real beamline images augmented for robustness, the model achieves near-perfect accuracy and millisecond-level inference speed, enabling real-time monitoring. The *FastCS* framework (Yendell, 2026 ▸) is control-system agnostic, so the same CNN codebase integrated here with *Experimental Physics and Industrial Control System* (*EPICS*) (Dalesio *et al.*, 2019 ▸) could be deployed at facilities using *TANGO* (Chaize *et al.*, 1999 ▸) with no changes. This advancement significantly reduces the risk of equipment collisions and supports fully automated sample changes on complex beamline endstations.

## 2. Methods and results

### 2.1. Operational dataset and labelling

**Figure 1:**
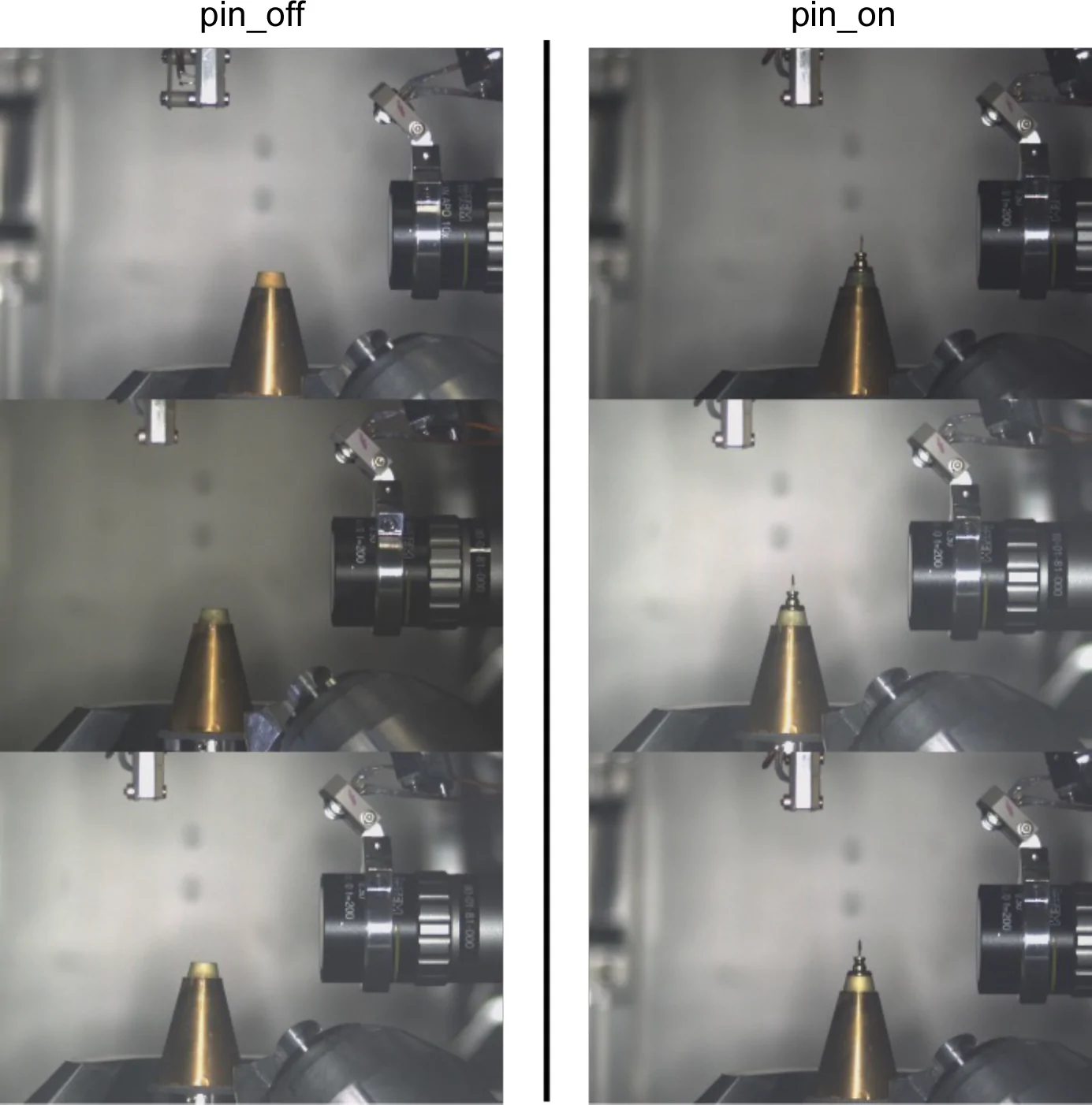
Representative cropped images from the I23 sample environment camera, illustrating the natural variability present in the operational dataset, including changes in illumination, minor camera repositioning, and the presence or absence of ancillary equipment in the field of view. Frames were collected during routine sample changes; class labels were assigned automatically from operator input and then manually verified before training.

During routine sample changes on beamline I23, camera frames were captured automatically whenever the control system requested operator confirmation of sample presence. This produced a large operational dataset with minimal additional curation effort: labels were assigned automatically from routine operator input and subsequently verified before training. The dataset spans two calendar years and naturally captures variability in illumination, minor camera repositioning and the presence or absence of ancillary equipment in the field of view (Fig. 1 ▸).

Each image was automatically assigned to pin_on or pin_off depending on input from the operator during routine sample change. Before training, images were manually checked and verified to belong to the assigned class. The camera model was an Allied Vision Manta G-125C with a Tamron 23FM25SP lens. Typical exposure settings are 0.03 s, gain = 2.0. A total of 4583 pin_off and 4170 pin_on images were collected (8753 in total). Of these, 875 images were set aside as a held-out test set, physically separated and never accessed by any training, tuning or model-selection script; they were used only for the final evaluation in Section 2.5 below. The remaining 7878 images were used for model development under a 70/30 train/test split (5515 training, 2363 test), the latter forming the evaluation set for batch-size comparison (Section 2.3).

### 2.2. Pre-processing and augmentation

Frames were centrally cropped by 100 pixels from each border to focus on the goniometer region, then resized by bilinear interpolation to 218 × 152 pixels (RGB) (20% linear resolution). Training-time augmentations (Shorten & Khoshgoftaar, 2019 ▸) comprised random rotation (±3°, fill_mode = reflect), translation (±5% of width/height, fill_mode = reflect), brightness/contrast adjustments (±40%) and additive Gaussian noise (σ = 1–3% of dynamic range), applied in the order geometric → photometric → noise.

### 2.3. Model architecture and training

A compact CNN for binary classification (pin_on versus pin_off) was trained using *keras* (Version 3.13.1; Chollet, 2015 ▸), *keras-tuner* (Version 1.4.8; O’Malley *et al.*, 2019 ▸) and *TensorFlow* (Version 2.20.0; Abadi *et al.*, 2016 ▸). To identify suitable hyperparameters, we used *keras-tuner* with the *Hyperband* search strategy (Li *et al.*, 2018 ▸). The search space comprised conv1 ∈ [16, 64] (step 8), conv1_2 ∈ [24, 96] (step 8), conv2 ∈ [16, 64] (step 8), conv2_2 ∈ [24, 96] (step 8), dense_units_1 ∈ [64, 256] (step 32), dense_units_2 ∈ [32, 128] (step 32) and learning_rate ∈ [5 × 10^−4^, 1 × 10^−3^, 2 × 10^−3^].

**Figure 2:**
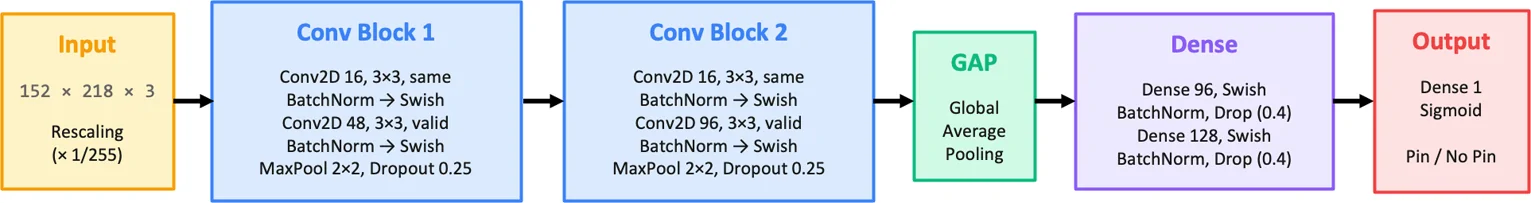
Schematic diagram of the *GoniOwl* CNN used for binary classification of sample-pin presence (pin_on versus pin_off) from RGB camera images (218 × 152 pixels). The network takes rescaled pixel values as input and comprises two convolutional stages, each containing two 3 × 3 convolutional layers with batch normalization and swish activation, followed by 2 × 2 max-pooling and dropout. The convolutional backbone is followed by global average pooling, two dense layers, and a sigmoid output neuron for binary classification. The architecture shown corresponds to the model selected in this study; the exact model definitions and training configuration are provided in the GitHub repository.

The final architecture (Fig. 2 ▸) comprised an input layer for 218 × 152 × 3 RGB images with pixel-value rescaling (division by 255) followed by two convolutional stages. The first stage consisted of a 3 × 3 convolution with 16 filters and same padding, batch normalization and swish activation, followed by a 3 × 3 convolution with 48 filters and valid padding, batch normalization and swish activation. This was followed by 2 × 2 max-pooling (stride 2) and dropout (rate 0.25). The second stage comprised a 3 × 3 convolution with 16 filters and same padding, batch normalization and swish activation, followed by a 3 × 3 convolution with 96 filters and valid padding, batch normalization and swish activation, again followed by 2 × 2 max-pooling (stride 2) and dropout (rate 0.25). The convolutional backbone was followed by global average pooling, then two fully connected layers of 96 and 128 units, each with swish activation, batch normalization and dropout (rate 0.4). The network terminated in a single sigmoid output neuron (cast to float32) for binary classification (Ioffe & Szegedy, 2015 ▸; Ramachandran *et al.*, 2017 ▸). The model contained 50513 trainable parameters and 800 non-trainable parameters with a file size of approximately 200 kB. Training used *Adam* (Kingma & Ba, 2015 ▸) (initial learning rate 0.001) with binary cross-entropy loss and a batch size of 16. The training data were shuffled at the start of each epoch (buffer size 1000, reshuffled each iteration). Learning-rate reduction (Reduce­LROnPlateau) and early stopping were configured to monitor the validation area under the receiver operating characteristic curve (AUC; maximization) rather than validation loss; on this small nearly balanced dataset (∼53%/47%) we found validation loss to be an unstable monitoring signal (see below), whereas validation AUC was threshold-independent and empirically stable. Random seeds were fixed. The small input size also allows training on low-end hardware.

To justify the choice of batch size, we independently retrained and hyperparameter-tuned the model at each batch size from 2 to 64, running a separate *keras-tunerHyperband* search per batch size, and trained five random seeds at each. Every resulting model was evaluated using the deployment-style confidence-gating policy (Section 2.5) on the 2363-image test fold of the 70/30 development split; the deployed model was subsequently assessed on the separate 875-image held-out set (Section 2.5), which no training or model-selection step ever accessed. Results of the batch-size comparison are summarized in Table 1 ▸, and in Fig. S1 of the supporting information. Training exhibited a seed-dependent instability in which a minority of runs collapsed to the majority-class baseline (accuracy ∼53%); this occurred across the batch-size range rather than only with small batches, motivating both the use of multiple seeds and the selection of the deployed model against the test set prior to use. Among the runs that trained successfully, very small batches (2 and 4) were the least reliable, while batch sizes of 8–24 gave the lowest error rates, with a mean of two false negatives, the safety-relevant error mode in which a present pin is missed, on the test set. False negatives increased at the largest batch sizes tested. We therefore selected a batch size of 16 for the deployed model, which lay within the best-performing range and trained reliably across seeds.

Training was performed on a single GPU (A100 ∼5 min, RTX 2000 Ada Generation ∼8 min) for up to 100 epochs. Validation AUC reached ∼1.0 within the first ∼15 epochs and validation accuracy stabilized at ∼100% from epoch 22 onward (Fig. S2 of the supporting information); the epoch-30 checkpoint was used for deployment. Training-set accuracy at this point was ∼99.6%.

The choice to monitor validation AUC rather than validation loss was necessary for stable training on this dataset. Because the validation set is small and the two classes are nearly balanced, the batch-normalization running statistics follow a slightly different trajectory under per-epoch shuffling, so the model’s calibration at the 0.5 decision threshold varies between epochs. This produced large epoch-to-epoch swings in validation loss and thresholded validation accuracy, with validation loss spiking by one to two orders of magnitude between consecutive epochs, even though the validation AUC remained stable at ∼0.998 throughout. When the learning-rate scheduler and early stopping monitored validation loss, these transient spikes were indistinguishable from a genuine plateau, prematurely decaying the learning rate and leaving the model at the majority-class baseline while training accuracy continued to rise. This apparent collapse was an artefact of the monitoring metric, not of model capacity. Switching to AUC-based monitoring yielded smooth convergence. A separate earlier issue, in which the data pipeline cached frames after augmentation and so froze the per-epoch ordering and augmentation across epochs, produced anomalous curves in which validation metrics exceeded training metrics; restructuring the pipeline to cache raw frames before shuffling and augmenting (cache → shuffle → augment → prefetch) restored the expected training dynamics. We note that, for tf.data.Dataset inputs, passing shuffle = True to the *keras*fit() method has no effect; shuffling must be applied within the dataset pipeline itself.

The presented architecture may differ if the hyperparameter search is rerun. Exact code and configuration files are provided in the GitHub repository (https://github.com/DiamondLightSource/GoniOwl).

### 2.4. Control system integration and decision policy

**Figure 3:**
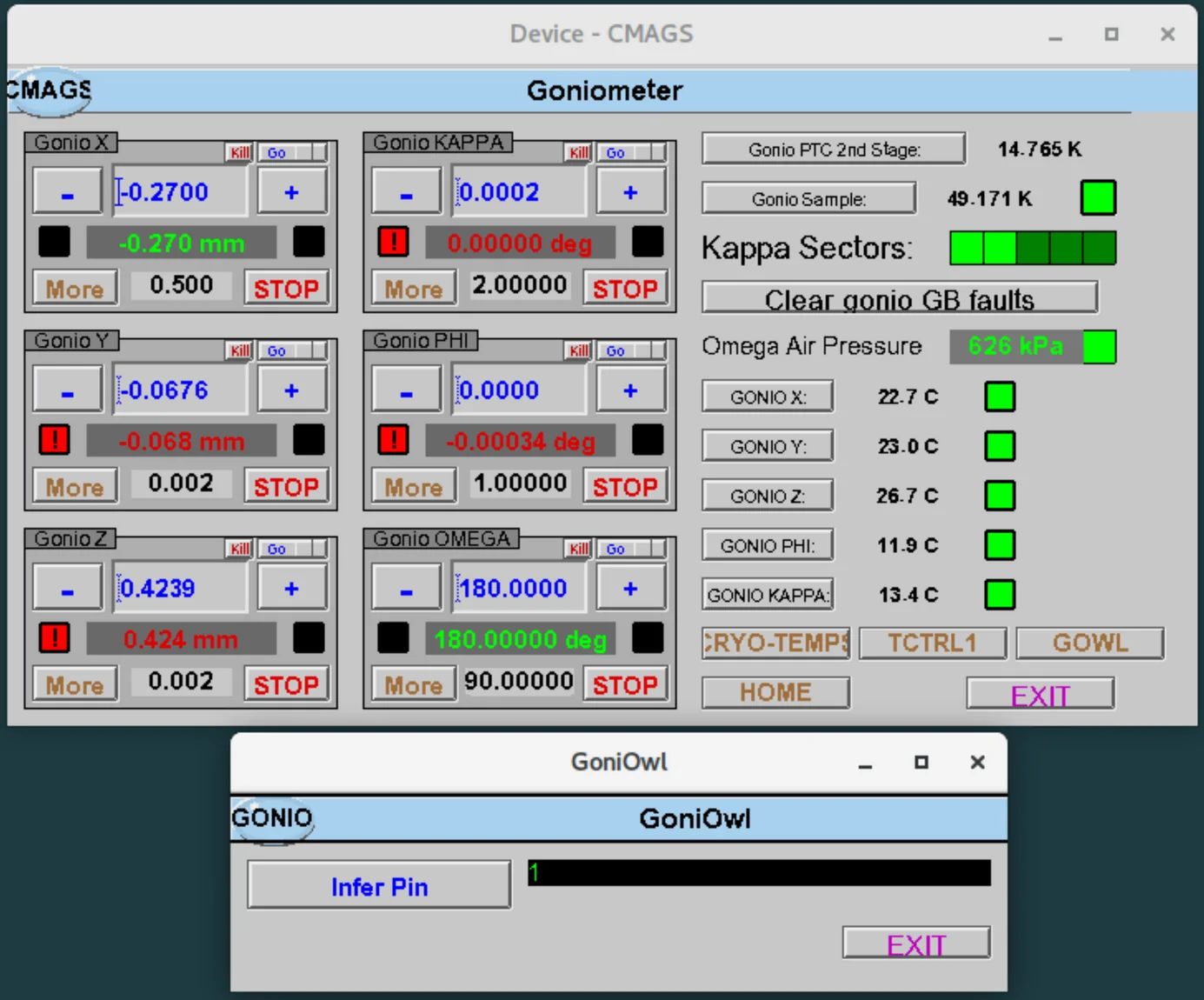
*EPICS* extensible display manager (EDM) screen for *GoniOwl*. The interface provides manual submission of an inference request from the sample-environment camera and displays the returned state code: 1 for pin_on, 2 for pin_off and 3 for undetermined. The undetermined state is used when a frame fails the exposure pre-check or when the CNN prediction falls below the confidence threshold required for autonomous operation.

At decision points in the sample-change sequence, an exposure pre-check identifies over/under-exposed frames (classified as ‘undetermined’) based on average intensity. The threshold values were determined by analysing the average intensity distribution of all training images, which have natural lighting variation present. The CNN then classifies valid frames. Model confidence scores are gated using a two-sided threshold: a prediction is treated as confident only when max(*s*, 1 − *s*) > τ with τ = 0.85, where *s* is the predicted probability of pin_on; intermediate scores are routed to the fail-safe undetermined state. We selected τ by re-running inference across all collected frames and binning predictions by confidence, then manually reviewing cases near the decision boundary to determine where false positives first emerged. False positives began to occur at approximately 0.80 confidence, so we adopted a more conservative operating point (τ = 0.85) to provide headroom against operational variability and to ensure uncertain cases are routed to the fail-safe ‘undetermined’ path rather than triggering autonomous motion (Geifman & El-Yaniv, 2017 ▸). Low-confidence predictions and over/under-exposed frames trigger a fail-safe: halt motion and request operator confirmation. Confident predictions are compared with sample tracking status; if consistent, the sample loading/unloading script continues. Integration was implemented using a Kubernetes-hosted *FastCS* Python input/output controller (IOC) publishing *EPICS* process variables (PVs) and reading from the camera stream on demand; inference ran on the I23 Kubernetes server (CPU), with median latency in the millisecond range (∼13 ms). Inference is synchronous relative to motion control. An extensible display manager (EDM) screen is provided to show the inference state (Fig. 3 ▸).

### 2.5. Evaluation protocol and classification performance

Model performance was assessed by offline inference on a held-out test set and on the full training set, using the same confidence-gating logic as in deployment. Metrics reported here correspond to the epoch-30 checkpoint of the batch-size-16 training run.

A prediction is treated as confident only when the model’s output score lies in the outer tails of the sigmoid range, specifically when max(*s*, 1 − *s*) > τ with τ = 0.85, where *s* is the predicted probability of pin_on. Scores in the intermediate band (0.15 ≤ *s* ≤ 0.85) are assigned to the fail-safe un­determined state and excluded from automated decisions. This mirrors the operational controller exactly.

**Figure 4:**
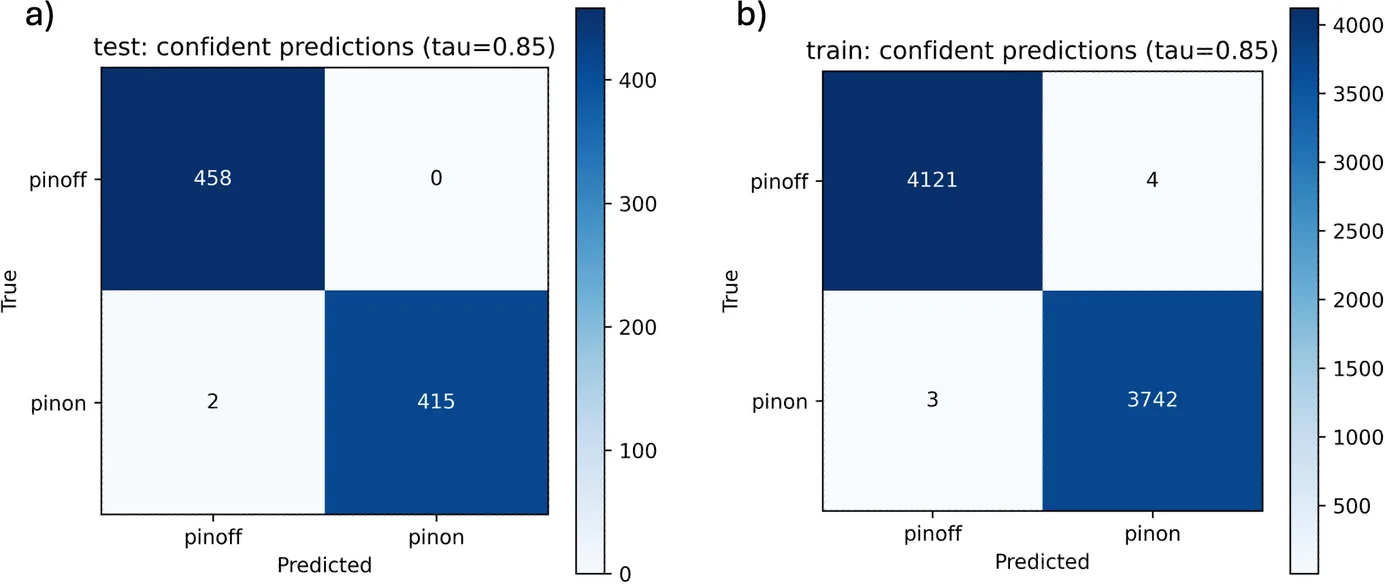
Confusion matrices for the deployed *GoniOwl* model (batch size 16, epoch-30 checkpoint) using deployment-style offline inference with two-sided confidence gating [confident if and only if max(*s*, 1 − *s*) > 0.85]. (*a*) The held-out test set and (*b*) the training set. Rows indicate true labels, columns predicted labels; class order is [pin_off, pin_on]. Predictions in the intermediate band were assigned to the fail-safe undetermined state and excluded from the matrices (none in the test set, eight in the training set).

On the held-out test set (875 images), every prediction was confident (coverage = 100.0%) and the model achieved 99.77% accuracy (873 of 875 correct). The two errors were pin_on frames classified as pin_off; there were no pin_off errors and no undetermined frames. The confusion matrix is shown in Fig. 4 ▸(*a*).

On the full development set (7878 images, comprising the training and test folds), re-evaluated to quantify residual error and confidence-gating behaviour on data seen during development (the model was trained with online augmentation and so never saw pixel-identical frames), the model produced 7870 confident predictions (coverage = 99.90%), of which 99.91% were correct (7863 of 7870). Eight frames were assigned to the undetermined state. The confusion matrix is shown in Fig. 4 ▸(*b*). A summary is given in Table 2 ▸.

Frames flagged as undetermined by the confidence gate were rare (none in the test set, eight in the training set), supporting the use of a conservative fail-safe policy for collision prevention. Uncertain cases are infrequent, but when they occur they are automatically removed from autonomous decision-making and handled via operator confirmation.

### 2.6. Deployment evidence and disagreement-audit loop

*GoniOwl* was deployed as a shadow method of assessing the sample environment state from 14 July 2025 to 23 February 2026. This meant the usual histogram, sample tracking and human input methods were used, and *GoniOwl* was run in the background to assess competence without directly affecting operations. Using independently verified labels as ground truth, the histogram method achieved 96% accuracy, primarily failing under occlusions and illumination changes; operator confirmations achieved 96% accuracy, with failures attributed to lapses in concentration and mis-typing; the CNN achieved 99% accuracy while assigning the remaining 1% to the fail-safe ‘undetermined’ state. No collisions occurred.

To track whether newer model versions resolve operationally critical mistakes, we implemented a two-stage retrospective-review workflow operating on deployment logs. First, a collector script scans the sample-change event log and copies every frame where the CNN decision disagreed with the operator confirmation and/or the legacy histogram method into a dedicated review directory. Each copied image is named with its event metadata (event ID, timestamp, original probabilities/decisions, exposure flag). This isolates all disagreement cases accumulated during background deployment for later offline review.

**Figure 5:**
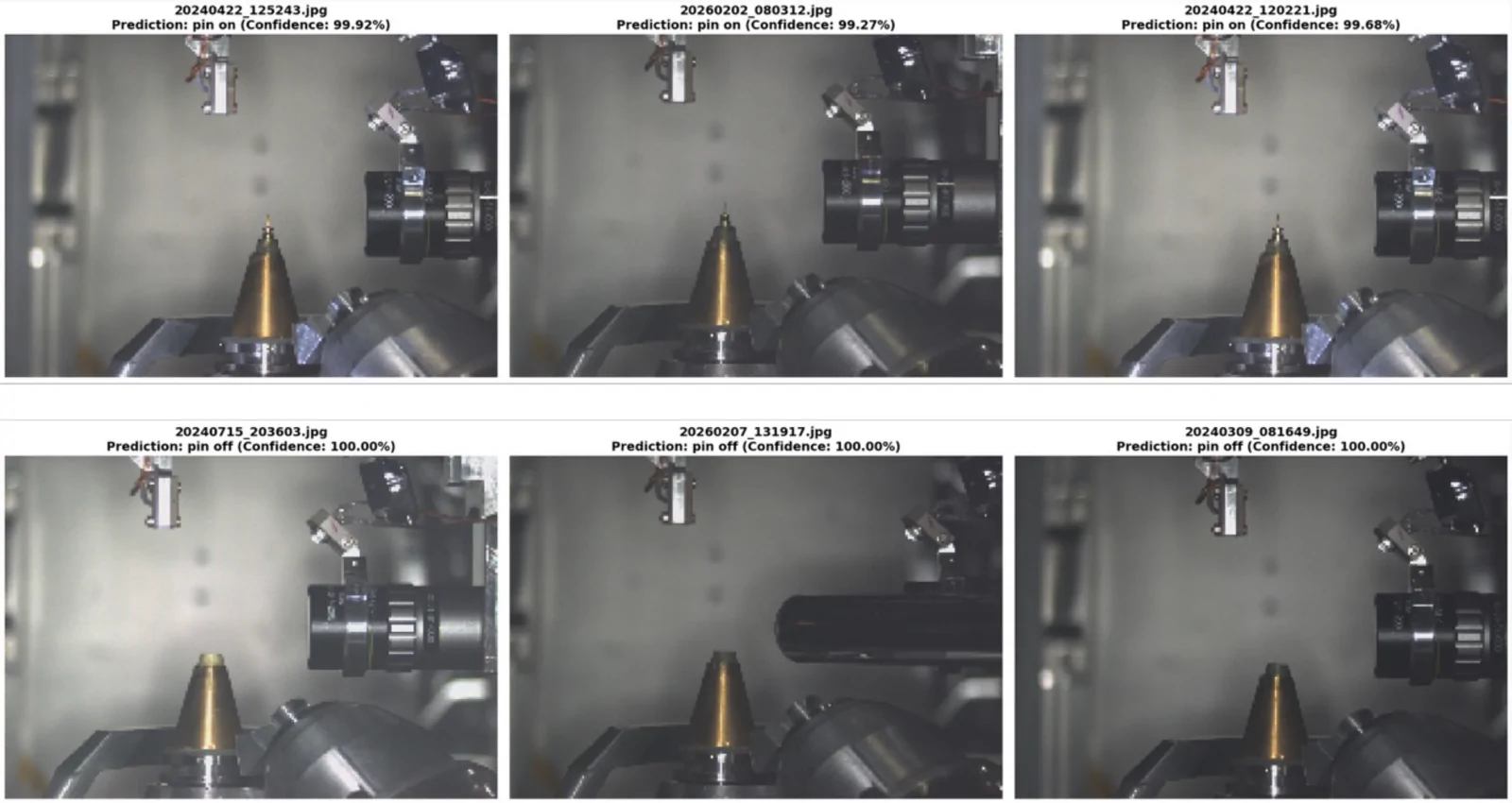
Examples from the disagreement-audit workflow, showing images that had previously disagreed with operator confirmation and/or the legacy histogram method, re-evaluated using the latest *GoniOwl* model. For each frame, the overlaid header reports the model probability and the corresponding decision at the operational threshold.

Second, an annotator script loads the latest trained CNN checkpoint and re-runs inference over every image in the review directory, outputting, for each input frame, a composite image that places the original frame below an overlaid header bar containing the latest model’s probability and binary decision (threshold τ) as shown in Fig. 5 ▸. This creates a visually scannable gallery for rapid expert triage of previously problematic cases. Because the training procedure uses randomized geometric, photometric and noise augmentations, the model has not directly seen these exact deployment frames during training. This provides a largely independent retrospective check, although the deployment frames remain correlated with the training data, so it reduces rather than removes evaluation bias.

This is a closed-loop monitoring strategy for machine learning model inference. During the formal shadow-mode deployment period (14 July 2025 to 23 February 2026), there were 305 disagreements between *GoniOwl* and human input. Re-running inference with the latest model trained on data up to 27 January 2026, 300 of 305 disagreements are correctly predicted with high confidence by *GoniOwl* and the remaining five were flagged as undetermined, largely due to the high confidence (85%+) required for determining state. Re-running the model was necessary because the model has been constantly changed since 2024 as this was an active research project.

### 2.7. Comparative approach: object detection

We evaluated transfer-learned object detection (*e.g.Faster R-CNN*) but observed sporadic false positives from background reflections and a higher labelling cost compared with folder-level class labelling (Ren *et al.*, 2015 ▸). Given safety requirements, the binary-state classifier offered higher operational reliability, with minimal curation overhead and streamlined retraining on up-to-date data. This was a qualitative evaluation of a different machine learning approach. If additional states at the sample position were to be considered, for example fluorescence detector position or tomography camera position, then this approach would be revisited.

## 3. Discussion

*GoniOwl* shows that a small instrument-specific image classifier can provide a practical and reliable software safeguard for automated beamline operation. In contrast to histogram rules, the CNN is resilient to operational variability, such as changes in illumination, small camera shifts, reflections and partial occlusions, because it is trained on a large set of real images that span this operational variability. Additional augmentation helps ensure the model does not just memorize the exact appearance of the scene under one set of acquisition conditions, but instead learns invariant features that remain valid under operational variability. The resulting high accuracy and low latency make the approach suitable for time-critical decisions in automated sample-changing sequences, where preventing costly collisions is the primary objective.

An earlier iteration of *GoniOwl* used a four-class design, adding explicit light and dark categories alongside pin_on and pin_off so that over- and under-exposed frames would be handled by the network itself rather than by a separate pre-check. In practice this model classified less reliably than the binary classifier, and it carried additional overhead: the light and dark classes had to be populated by deliberately generating over- and under-exposed images, increasing labelling and curation effort for little operational benefit. We therefore adopted the two-class design and delegated exposure handling to a deterministic pre-check based on average frame intensity, which is simpler, more transparent and easier to tune than an additional learned class.

The exposure pre-check and confidence gating (τ = 0.85) explicitly separate ‘known’ from ‘unknown’: frames that are invalid or predictions that are insufficiently confident are treated as undetermined and routed to a fail-safe pause with operator confirmation. This decision policy reduces the risk of incorrect autonomous actions, while keeping the un­determined rate low enough that it does not interfere with routine beamline operation. The combination of conservative gating and millisecond-scale inference provides an additional layer of protection that complements existing safeguards such as sample tracking and geometric constraints.

The deployment strategy further strengthens the reliability of the system. Running the model in a shadow mode provides an operationally realistic assessment against legacy logic and human confirmations without adding risk to the beamline hardware. The disagreement-audit workflow then turns the most informative failure modes into a curated stream of review material, enabling targeted retraining and verification that new model versions resolve previously problematic cases or, where appropriate, conservatively abstain. This closes an important loop for complex instruments where conditions naturally evolve, and where the practical definition of robustness is continued performance under changing and imperfectly controlled environments.

There are, however, limitations. The model is trained on a specific camera view and mechanical context; substantial changes to optics, illumination hardware or geometry can introduce distribution shifts that require additional data collection and retraining. Labels derived from operator confirmations can also contain occasional errors, so ongoing curation and periodic verification remain important. Finally, while the binary classifier is well matched to the collision-prevention requirement, extending the approach to additional states (*e.g.* nearby device positions or more complex scene configurations) may warrant revisiting multi-class classification or object detection. Either option would come at the cost of higher labelling effort and would require care to control false positives in reflective environments.

## 4. Conclusion

We have developed *GoniOwl*, a compact CNN-based classifier that determines sample pin presence/absence from routine beamline camera images and integrates directly into automated sample-handling workflows to reduce collision risk. By training on a large manually verified set of operational images and using targeted augmentations, the model remains robust to changes such as varying illumination levels, small viewpoint shifts, reflections and partial occlusions.

For machine-protection operation, *GoniOwl* is deployed with a conservative decision policy: frames that are over/under-exposed or predictions that fall below a confidence threshold are treated as undetermined, triggering a fail-safe pause and operator confirmation rather than an automated action. Integrated into the control system, the approach provides fast on-demand inference suitable for real-time decision points, and supports unattended high-throughput operation.

Finally, we introduced a disagreement-audit workflow that automatically collects and re-evaluates cases where the model and legacy logic or human confirmation diverge, enabling systematic review and iterative improvement as instrument conditions evolve. The combination of robust classification, fail-safe gating and closed-loop monitoring demonstrated here is not specific to I23; any beamline or instrument environment where a camera provides a stable view of operationally relevant states can adopt the same approach. The open-source code, trained model and reproducible training scripts in the *GoniOwl* repository allow a custom version to be deployed with minimal prerequisites, requiring only a labelled image set from the target instrument.

## Supplementary Material

Figures S1 and S2. DOI: 10.1107/S1600577526007277/yn5136sup1.pdf

Raw training images for GonioOwl: https://doi.org/10.5281/zenodo.17047675

## Figures and Tables

**Table 1:** Effect of batch size on *GoniOwl* performance across multiple random seeds Each batch size was independently hyperparameter-tuned (separate *keras-tuner Hyperband* search) and then retrained from five random seeds, with every resulting model evaluated on the 2363-image test fold of the 70/30 development split (1254 pin-absent, 1109 pin-present) using the deployment-style confidence-gating policy [confident if and only if max(*s*, 1 − *s*) > 0.85]. The deployed model’s performance on the separate 875-image held-out set, never seen during training, tuning or model selection, is reported in Section 2.5 and Table 2. For each batch size we report the number of seeds that trained successfully versus those that collapsed to the majority-class baseline (accuracy ∼53%) and, computed over the successful runs only, the mean test accuracy and the mean and range of false negatives (missed pin-present frames), the error mode most relevant to collision prevention. Training instability occurred across the batch-size range rather than just for small batches, motivating the use of multiple seeds and model selection against the test set prior to deployment. A batch size of 16 was adopted for the deployed model.

Batch size	Seeds trained / total	Mean accuracy (%)	Mean false negatives	False negative range	Mean total errors
2	0 / 5	–	–	–	–
4	2 / 5	99.68	4.5	2–7	7.5
8	4 / 5	99.82	2.25	2–3	4.2
12	4 / 5	99.82	2.0	2	4.2
16	4 / 5	99.82	2.0	2	4.2
24	4 / 5	99.82	2.0	2	4.2
32	4 / 5	99.76	3.5	2–7	5.8
46	5 / 5	98.37	36.4	2–109	38.4
64	4 / 5	99.71	4.5	2–11	6.8

**Table 2:** Summary of deployment-style offline inference performance for *GoniOwl* on the held-out test set and training set, using the operational two-sided confidence-gating policy [confident if and only if max(*s*, 1 − *s*) > 0.85] Reported values are the total number of images, the number of confident predictions, the number assigned to the fail-safe undetermined state, the corresponding coverage, and the accuracy on the confident subset.

Dataset	Total images (*N*)	Confident predictions (*n*)	Undetermined (*N* − *n*)	Coverage (%)	Correct among confident	Accuracy on confident subset (%)
Test set	875	875	0	100.00	873 / 875	99.77
Training set	7878	7870	8	99.90	7863 / 7870	99.91

## Data Availability

The source code, trained model, instructions and reproducible scripts are available at https://github.com/DiamondLightSource/GoniOwl (release tag v1.0, licence: Apache 2.0). The curated image dataset is archived at Zenodo (Orr & Pearl, 2025 ▸).

## References

[bb1] Abadi, M., Barham, P., Chen, J., Chen, Z., Davis, A., Dean, J., Devin, M., Ghemawat, S., Irving, G., Isard, M., Kudlur, M., Levenberg, J., Monga, R., Moore, S., Murray, D. G., Steiner, B., Tucker, P., Vasudevan, V., Warden, P., Wicke, M., Yu, Y. & Zheng, X. (2016). *Operating Systems Design and Implementation*, pp. 265–283. Savannah, Georgia, USA.

[bb2] Chaize, J.-M., Götz, A., Klotz, W.-D., Meyer, J., Perez, M. & Taurel, E. (1999). *Proceedings of the 7th International Conference on Accelerator and Large Experimental Physics Control Systems (ICALEPCS1999)*, 4–8 October 1999, Trieste, Italy, pp. 475–479.

[bb3] Chollet, F. (2015). *keras*, https://keras.io.

[bb4] Dalesio, L. R., Johnson, A. N. & Kasemir, K.-U. (2019). *Proceedings of the 17th International Conference on Accelerator and Large Experimental Physics Control Systems (ICALEPCS2019)*, 5–11 October 2019, New York, USA.

[bb5] Duman, R., Orr, C. M., Mykhaylyk, V., El Omari, K., Pocock, R., Grama, V. & Wagner, A. (2021). *J. Vis. Exp.* **170**, e62364. 10.3791/62364 33970149

[bb6] Geifman, Y. & El-Yaniv, R. (2017). *Proceedings of the 31st International Conference on Neural Information Processing Systems (NIPS2017)*, 4–9 December 2017, Long Beach, California, USA, pp. 4885–4894.

[bb7] Grama, V. & Wagner, A. (2016). *Proceedings of 9th International Workshop on Mechanical Engineering Design of Synchrotron Radiation Equipment and Instrumentation (MEDSI2016)*, 11–16 September 2016, Barcelona, Spain, pp. 267–271. WEAA04.

[bb8] Ioffe, S. & Szegedy, C. (2015). *Int. Conf. Mach. Learn.* **37**, 448–456.

[bb10] Kingma, D. P. & Ba, J. (2015). *Proceedings of the Third International Conference on Learning Representations (ICLR2015)*, 7–9 May 2015, San Diego, A, USA (*arXiv*:1412.6980).

[bb11] Li, L. S., Jamieson, K., DeSalvo, G., Rostamizadeh, A. & Talwalkar, A. (2018). *J. Mach. Learn. Res.* **18**, 1–52.

[bb12] Nurizzo, D., Bowler, M. W., Caserotto, H., Dobias, F., Giraud, T., Surr, J., Guichard, N., Papp, G., Guijarro, M., Mueller-Dieckmann, C., Flot, D., McSweeney, S., Cipriani, F., Theveneau, P. & Leonard, G. A. (2016). *Acta Cryst.* D**72**, 966–975. 10.1107/S205979831601158X PMC4973212 27487827

[bb13] O’Hea, J. D., Burt, M., Fisher, S., Jones, K. M. J., McAuley, K. E., Preece, G. & Williams, M. A. (2017). *Proceedings of the 16th International Conference on Accelerator and Large Experimental Control Systems (ICALEPCS2017)*, 12–16, October 2017, Barcelona, Spain, pp. 1919–1922.

[bb229] O’Malley, T., Bursztein, E., Long, J., Chollet, F., Jin, H. & Invernizzi, L. (2019). *KerasTuner*, https://github.com/keras-team/keras-tuner.

[bb14] Orr, C. M. & Pearl, D. (2025). *Raw training images for *GoniOwl**, https://zenodo.org/records/17047675.

[bb15] Ramachandran, P., Zoph, B. & Le, Q. V. (2017). *arXiv*:1710.05941.

[bb16] Rees, N. P., Denison, P. N. & Cobb, T. M. (2007). *Proceedings of the 11th International Conference on Accelerator and Large Experimental Physics Control Systems (ICALEPCS2007)*, 15–19 October 2007, Knoxville, Tennessee, USA, pp. 108–110.

[bb17] Ren, S. Q., He, K. M., Girshick, R. & Sun, J. (2015). *Advances in Neural Information Processing Systems 28 (NIPS 2015)*, edited by C. Cortes, N. Lawrence, D. Lee, M. Sugiyama & R. Garnett, pp. 91–99 (*arXiv*:1506.01497).

[bb18] Shorten, C. & Khoshgoftaar, T. M. (2019). *J. Big Data* **6**, 60.

[bb9] Sulaiman Khail, W., Schnizer, P. & Ries, M. (2023). *19th International Conference on Accelerator and Large Experimental Physics Control Systems (ICALEPCS2023)*, Cape Town, South Africa, pp. 1594–1599.

[bb19] Wagner, A., Duman, R., Henderson, K. & Mykhaylyk, V. (2016). *Acta Cryst.* D**72**, 430–439. 10.1107/S2059798316001078 PMC4784674 26960130

[bb20] Yendell, G. (2026). *FastCS: Control System Agnostic Framework for Building Device Support in Python.*https://diamondlightsource.github.io/fastcs/main/index.html.

