# Supplementary material for: GoniOwl: convolutional neural network-based sample state detection for collision prevention on synchrotron beamlines
Source: J Synchrotron Radiat. 2026 Aug 7;33(Pt 5):1356–63. doi: 10.1107/S1600577526007277 (PMC13549329; doi:10.1107/S1600577526007277)
Supplement: Supplementary file 1 [file s-33-01356-sup1.pdf]

JOURNAL OF SYNCHROTRON RADIATION

Volume 33 (2026)

Supporting information for article:

GoniOwl: convolutional neural network-based sample state detection for collision prevention on synchrotron beamlines

Christian M Orr, James O'Hea and Armin Wagner

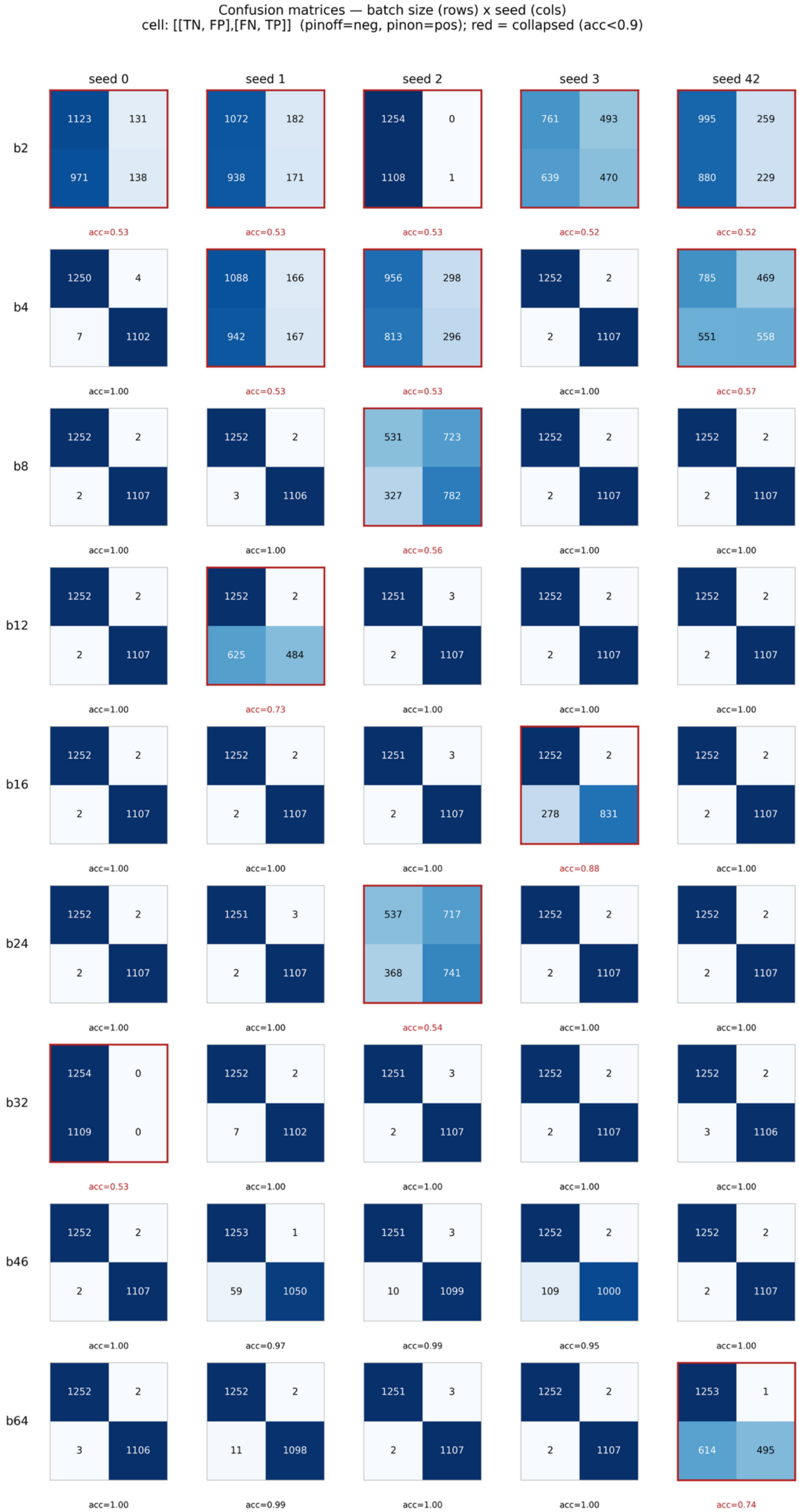
Confusion matrices — batch size (rows) x seed (cols)
cell: [[TN, FP],[FN, TP]] (pinoff=neg, pinon=pos); red = collapsed (acc<0.9)
seed 0
seed 1
seed 2
seed 3
seed 42
b2
1123 131 971 138 acc=0.53
1072 182 938 171 acc=0.53
1254 0 1108 1 acc=0.53
761 493 639 470 acc=0.52
995 259 880 229 acc=0.52
b4
1250 4 7 1102 acc=1.00
1088 166 942 167 acc=0.53
956 298 813 296 acc=0.53
1252 2 2 1107 acc=1.00
785 469 551 558 acc=0.57
b8
1252 2 2 1107 acc=1.00
1252 2 3 1106 acc=1.00
531 723 327 782 acc=0.56
1252 2 2 1107 acc=1.00
1252 2 2 1107 acc=1.00
b12
1252 2 2 1107 acc=1.00
1252 2 625 484 acc=0.73
1251 3 2 1107 acc=1.00
1252 2 2 1107 acc=1.00
1252 2 2 1107 acc=1.00
b16
1252 2 2 1107 acc=1.00
1252 2 2 1107 acc=1.00
1251 3 2 1107 acc=1.00
1252 2 278 831 acc=0.88
1252 2 2 1107 acc=1.00
b24
1252 2 2 1107 acc=1.00
1251 3 2 1107 acc=1.00
537 717 368 741 acc=0.54
1252 2 2 1107 acc=1.00
1252 2 2 1107 acc=1.00
b32
1254 0 1109 0 acc=0.53
1252 2 7 1102 acc=1.00
1251 3 2 1107 acc=1.00
1252 2 2 1107 acc=1.00
1252 2 3 1106 acc=1.00
b46
1252 2 2 1107 acc=1.00
1253 1 59 1050 acc=0.97
1251 3 10 1099 acc=0.99
1252 2 109 1000 acc=0.95
1252 2 2 1107 acc=1.00
b64
1252 2 3 1106 acc=1.00
1252 2 11 1098 acc=0.99
1251 3 2 1107 acc=1.00
1252 2 2 1107 acc=1.00
1253 1 614 495 acc=0.74

Figure S1 Held-out test-set confusion matrices for GoniOwl across batch sizes from 2 to 64, with each batch size independently hyperparameter-tuned (separate Keras-Tuner Hyperband search), using deployment-style offline inference with two-sided confidence gating (confident if and only if max(s, 1 − s) > 0.85). For each batch size, rows indicate true labels and columns predicted labels; class order is [pin_off, pin_on]. Predictions in the intermediate band were assigned to the fail-safe undetermined state and excluded from the matrices. Batch sizes of 2 and 4 fail to train reliably; 8–24 give the lowest error rates; larger batches show a gradual increase in misclassifications, including false negatives (missed pins), the error mode most relevant to collision prevention.

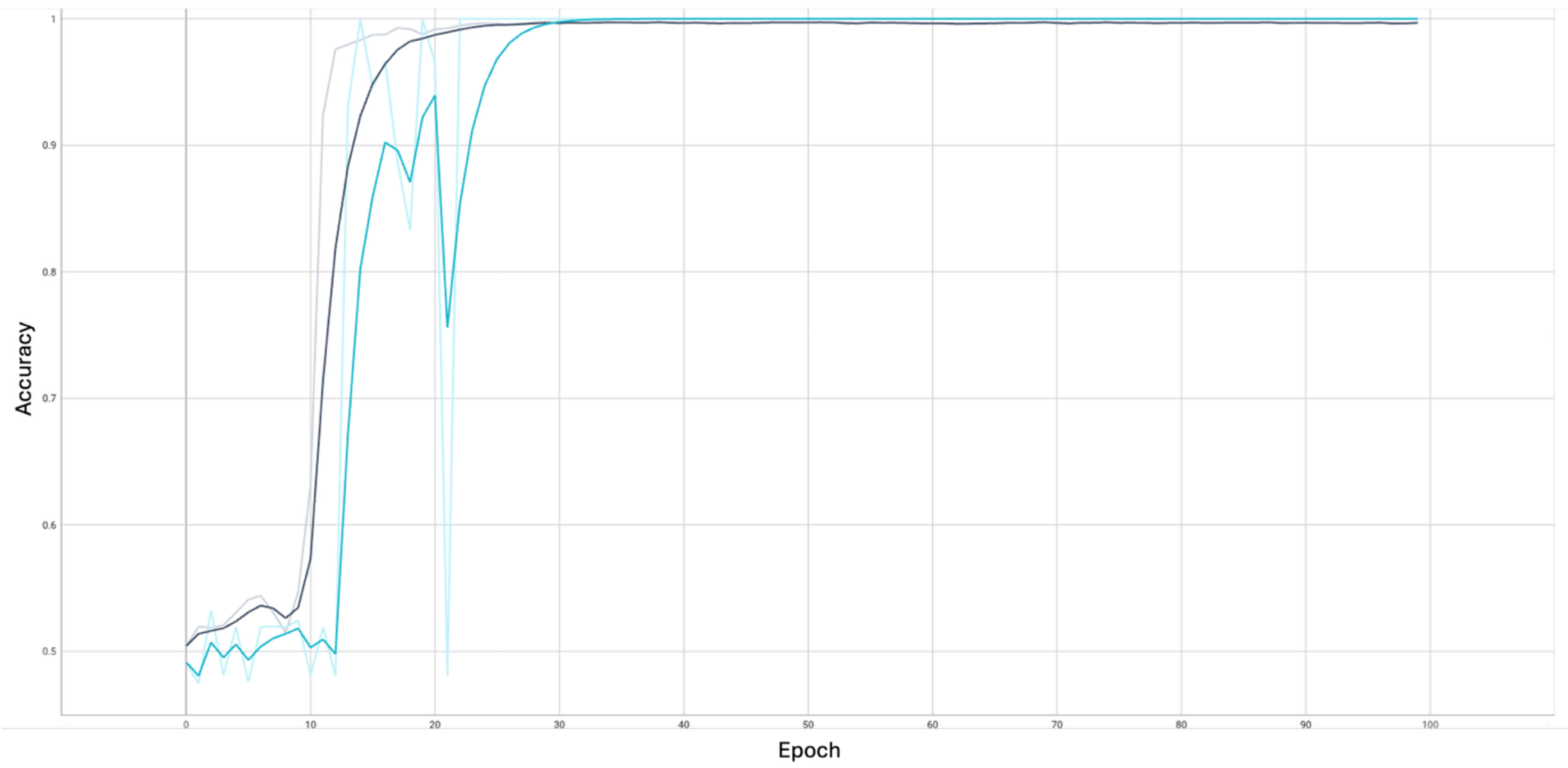

Figure S2 Training and validation accuracy versus epoch for the deployed GoniOwl model (batch size 16, best-performing seed), with the learning-rate schedule and early stopping monitoring validation AUC. Both curves rise from the near-balanced majority baseline (≈53%) and converge to near-perfect accuracy, with validation accuracy tracking training accuracy after the initial transient and remaining stable through to 100 epochs. The transient dips before convergence reflect epoch-to-epoch variation in batch-normalisation calibration at the 0.5 decision threshold on this small, near-balanced dataset; these affect the thresholded accuracy but not the validation AUC, which remained stable throughout (Section 2.3).
